# Interceptive Management of Ectopically Erupting Maxillary Canines With Rapid Maxillary Expansion and Clear Aligners: A Case Report

**DOI:** 10.1155/crid/3778979

**Published:** 2026-11-10

**Authors:** Mauro Lorusso, Gaetano Illuzzi, Michele Tepedino, Lorenzo Bartolommei, Daniela Lupini, Ettore Gaudiosi, Fariba Esperouz, Lucio Lo Russo, Domenico Ciavarella

**Affiliations:** ^1^ Department of Clinical and Experimental Medicine University of Foggia Foggia Italy unifg.it; ^2^ Department of Biotechnological and Applied Clinical Sciences Dental School of L′Aquila University of L′Aquila L′Aquila Italy univaq.it; ^3^ Private Practice Sansepolcro Italy; ^4^ Department of Medical Surgical and Health Sciences School of Dentistry University of Trieste Trieste Italy units.it

**Keywords:** case report, clear aligners, growing patients, impacted canines, sequential distalization

## Abstract

**Objective:**

Maxillary canine impaction represents one of the most common eruption disturbances in orthodontics after third molars. This case report describes the management of a 9.2‐year‐old growing male patient with bilateral ectopic eruption of the maxillary permanent canines, transverse maxillary deficiency, mesiorotation of the upper first molars, and bilateral Class II molar relationship. The patient was treated according to the BIG‐C concept (Biomechanical Interceptive Guidance of Canine Eruption)—a biomechanically guided interceptive approach combining rapid maxillary expansion, sequential distalization with clear aligners, and arch‐perimeter management.

**Methods:**

Radiographic changes were assessed on serial panoramic radiographs using the Ericson and Kurol criteria, including *α*‐angle and sector classification at baseline (T0), after RME (T1), and after sequential distalization (T2). Clinical and radiographic follow‐up was continued until eruption and progressive alignment of both permanent canines following interceptive treatment were observed (T3).

**Results:**

Both maxillary canines showed progressive radiographic improvement. The *α*‐angle decreased from 44.5° to 17.7° for Tooth 1.3 and from 47.1° to 18.7° for Tooth 2.3, corresponding to total reductions of 60.2% and 60.3%, respectively. Sector relationships improved to Sector 1 for both canines, and eruption occurred without surgical exposure or direct orthodontic traction.

**Conclusions:**

The combined interceptive protocol, including rapid maxillary expansion followed by sequential distalization with clear aligners, was associated with progressive *α*‐angle reduction, improvement in sector relationship, and eruption of both maxillary permanent canines in this case. The contribution of the individual treatment components cannot be determined from this single observation.

## 1. Introduction

Impaction of the maxillary permanent canine is one of the most common eruption disturbances in orthodontics and may lead to prolonged treatment, root resorption of adjacent incisors, surgical exposure, and complex orthodontic traction mechanics [1]. Early radiographic assessment is important for identifying ectopic canine eruption before complete impaction occurs [2]. According to the Ericson and Kurol criteria, prognosis is related primarily to canine inclination, vertical position, and the mesiodistal sector of the canine crown [2, 3].

Extraction of the primary canine has traditionally represented a common interceptive approach, although its effectiveness may be limited in cases with unfavorable canine position or reduced arch perimeter [4]. Recent evidence on palatally displaced canines supports the rationale that interceptive procedures should not be limited to space recovery through extraction but may also include strategies aimed at improving transverse development, maintaining arch perimeter, and preventing mesial migration of the upper first molars [5]. In particular, rapid maxillary expansion has been shown to increase the likelihood of spontaneous eruption of palatally displaced canines compared with untreated controls, and the expansion may be combined with deciduous canine extraction and/or molar anchorage control when arch‐perimeter preservation is required [6]. Mandibular arch morphology may also differ in patients with maxillary canine displacement and may provide complementary diagnostic information [7, 8].

More recently, clear aligner therapy has progressively expanded its indications in growing patients, including dentoalveolar expansion in mixed dentition, Class II correction, and sequential molar distalization [9, 10]. Nevertheless, current evidence on aligners for impacted or ectopically erupting maxillary canines remains limited and is mainly based on case reports or small clinical studies, often involving auxiliaries rather than pure aligner‐driven biomechanics [11]. Therefore, although the biological and biomechanical rationale for maintaining or increasing maxillary arch perimeter is supported by the literature, limited evidence is currently available regarding the use of sequential distalization with clear aligners as one component of interceptive protocols for ectopically erupting maxillary canines and its temporal association with changes in *α*‐angle and eruption trajectory.

The BIG‐C concept (Biomechanical Interceptive Guidance of Canine Eruption) does not introduce novel individual appliances or treatment modalities. Rather, its proposed novelty lies in the coordinated and sequential integration of established interceptive procedures including transverse correction, posterior space management, arch‐perimeter preservation, and digitally planned tooth movement within a single treatment framework specifically directed toward optimizing the eruptive environment of ectopically positioned maxillary canines. Therefore, the objectives of the present case report were to describe the application of the BIG‐C therapeutic concept in this individual patient, to evaluate radiographic changes according to the Ericson and Kurol criteria and to discuss the possible biomechanical, developmental, and treatment‐related factors associated with the observed clinical evolution.

## 2. Case Presentation

This study was conducted following the CARE guidelines [12]. Written informed consent for the patient′s participation and for the publication of this case report, including any clinical images and data, was obtained from the patient′s parents/legal guardians. The study was conducted in accordance with the principles of the Declaration of Helsinki and was approved by the Ethics Committee of the University of Foggia (Approval No. 92/CE/2025).

A growing male patient, aged 9.2 years, in mixed dentition and presenting with bilateral ectopic eruption of the maxillary permanent canines, was treated according to the BIG‐C therapeutic approach, which in this case consisted of rapid maxillary expansion followed by sequential distalization with clear aligners.

The radiographic and clinical progression was documented at the following time points:

• T0 (baseline, January 2023): initial diagnosis and baseline panoramic radiograph.
• T1 (November 2023): 10 months after baseline, following completion of rapid maxillary expansion and initiation of clear aligner therapy.
• T2 (March 2025): 16 months after T1 and 2 months after extraction of the primary canines, following completion of the active sequential distalization phase.
• T3 (March 2026): 12 months after T2, at the final radiographic follow‐up, when eruption and progressive alignment of both permanent canines were observed.

Radiographic measurements were performed according to the diagnostic and prognostic criteria originally described by Ericson and Kurol [2]. The *α*‐angle was defined as the angle formed between the long axis of the maxillary canine and the facial midline, which was identified on each panoramic radiograph as a vertical reference line passing through the midline of the nasal cavity. Sector classification was determined according to the mesiodistal position of the canine crown relative to the adjacent lateral incisor root. Buccopalatal position was not assessed because CBCT imaging was not performed, as three‐dimensional imaging was not considered clinically justified in this young patient because of the additional radiation exposure.

The quantitative longitudinal assessment focused on *α*‐angle and sector classification. Vertical canine position relative to the occlusal plane was monitored qualitatively on the serial panoramic radiographs but was not included as a quantitative outcome, because absolute linear measurements on panoramic images may be influenced by projection geometry and magnification differences between acquisitions.

Figure 1 illustrates the cephalometric tracing obtained at baseline, whereas Figure 2 illustrates the cephalometric tracing obtained after treatment. Table 1 shows the cephalometric variables at baseline and after treatment.

**Figure 1:**
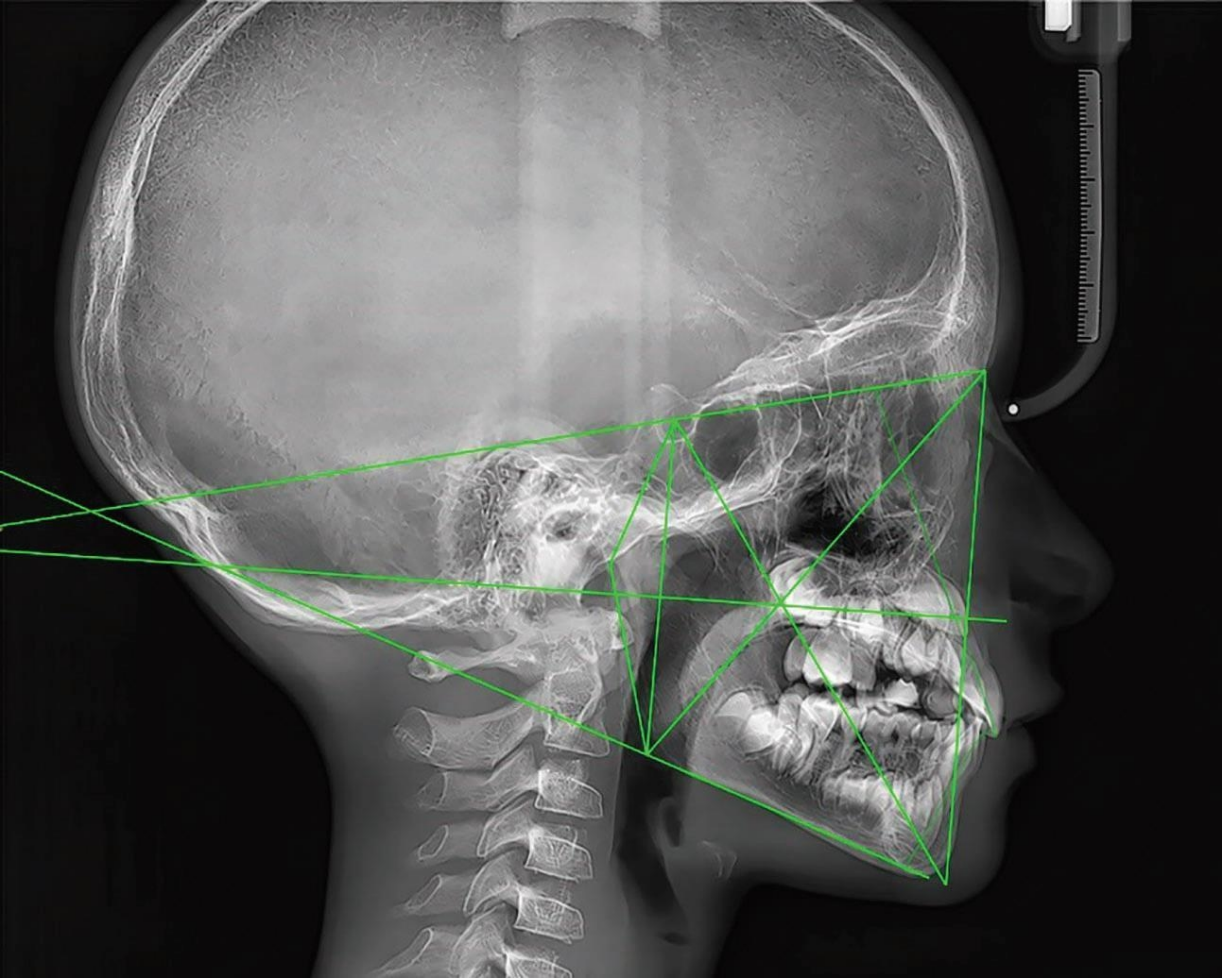
Pretreatment cephalometric tracing.

**Figure 2:**
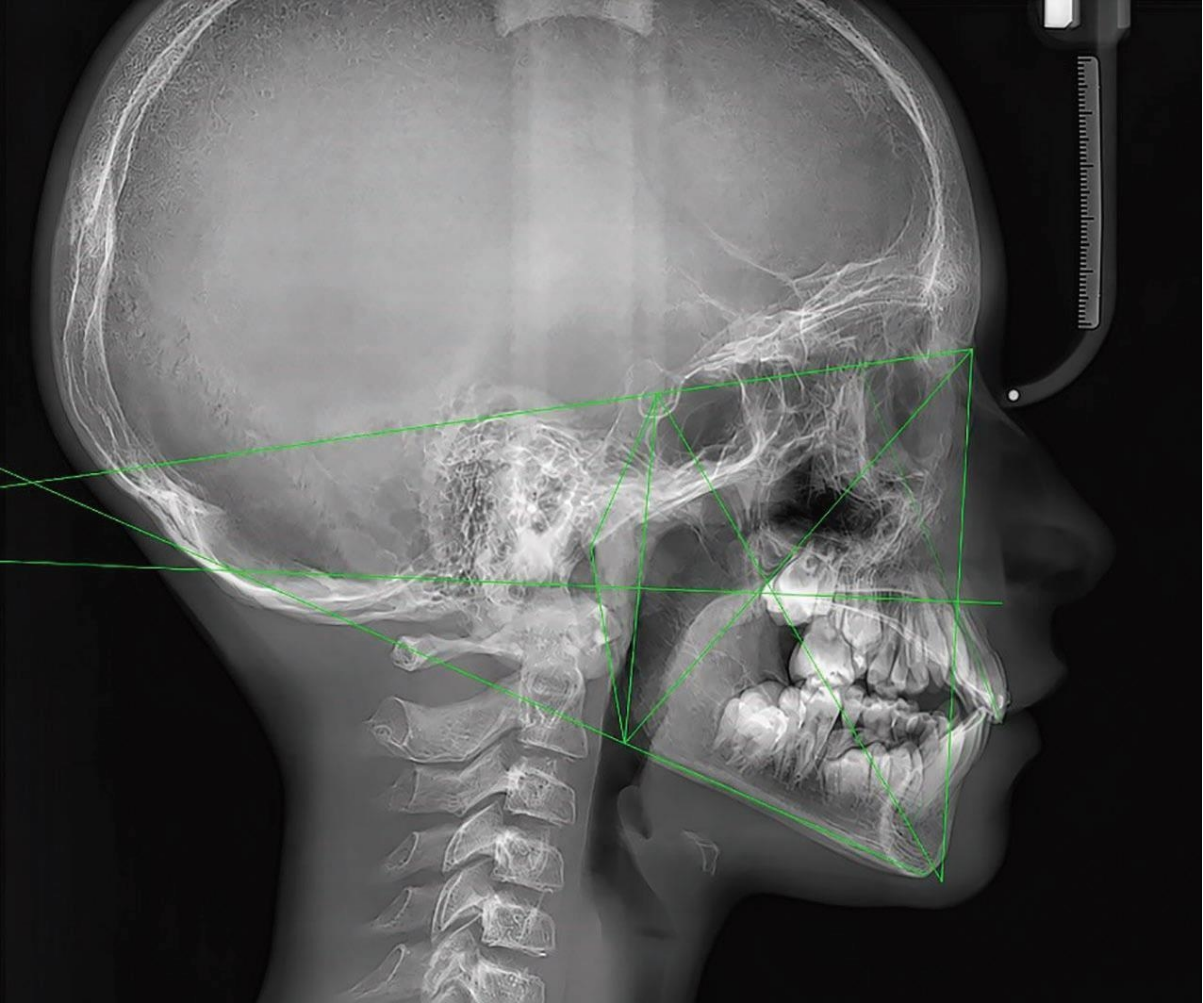
Posttreatment cephalometric tracing.

**Table 1:** Cephalometric variables at baseline and after treatment.

Cephalometric variable	Pretreatment	Posttreatment
Lower incisor to Go‐Gn	94.87°	102.51°
Upper incisor to SN	100.18°	103.52°
Upper gonial angle	52.1°	51.3°
Lower gonial angle	72.7°	72.3°
SNA angle	81.2°	82.3°
SNB angle	78.1°	80.0°
ANB angle	3.1°	2.3°
Mandibular plane angle to cranial base	32.9°	31.8°

To minimize random error, cephalometric variables were measured twice. For each variable, the random error was determined using Dahlberg′s formula, in which *d* corresponds to the difference between the two repeated measurements and *N* represents the number of radiographs analyzed [13]. The calculated random error ranged from 0.08 to 0.14 mm for linear variables and from 0.1° to 0.12° for angular variables. Measurements were repeated twice by the same operator at a 2‐week interval, and mean values were used for analysis in order to reduce measurement variability.

Table 2 presents the progressive reduction in *α*‐angle values and the corresponding changes in sector relationships across the different treatment phases. The largest numerical reduction in *α*‐angle was observed between T1 and T2, an interval during which sequential distalization, molar derotation, arch‐perimeter management, continued dental development, and subsequent extraction of the primary canines occurred. A progressive apical‐to‐occlusal advancement of both canine crowns was also evident on serial panoramic examination; however, vertical position was not quantitatively compared because no calibrated linear outcome had been predefined for this parameter.

**Table 2:** Differences in canine positions during treatment.

Tooth	T0 *α*‐angle	T0 sector	T1 *α*‐angle	T1 sector	T2 *α*‐angle	T2 sector
1.3	44.5°	S2	31.2°	S2	17.7°	S1
2.3	47.1°	S3	31.9°	S2	18.7°	S1

*Note:* T0, baseline; T1, after rapid maxillary expansion; T2, after extraction of the primary canines and completion of the active sequential distalization phase.

### 2.1. Diagnosis and Initial Assessment

Clinical examination revealed a transverse maxillary deficiency, bilateral Class II molar relationship, mesiorotation of the maxillary first permanent molars, reduced maxillary arch perimeter, and compromised mixed‐dentition anchorage because of posterior deciduous teeth that were decayed, close to exfoliation, or already missing. On pretreatment digital models, the posterior transverse interarch discrepancy (PTID), calculated as the difference between mandibular and maxillary intermolar widths at the level of the first permanent molars, was 4.0 mm, confirming a clinically relevant maxillary transverse deficiency.

Cephalometric analysis showed a skeletal Class I sagittal relationship (SNA, 81.2°; SNB, 78.1°; ANB, 3.1°) and a normodivergent vertical skeletal pattern, with a mandibular plane angle to the cranial base of 32.9°. The maxillary and mandibular incisor inclinations were 100.18° relative to SN and 94.87° relative to Go‐Gn, respectively. Overall, the cephalometric assessment did not identify a major sagittal or vertical skeletal discrepancy as a primary treatment concern; the principal skeletal component relevant to treatment planning was the transverse maxillary deficiency. Pretreatment extraoral and intraoral photographs are shown in Figure 3.

**Figure 3:**
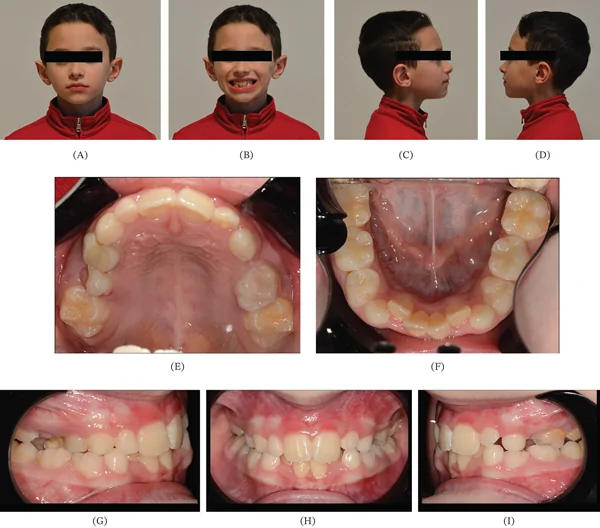
Pretreatment extraoral and intraoral photographs: (A) frontal view with lips at rest, (B) frontal view during smiling, (C) right profile view, and (D) left profile view. Intraoral photographs: (E) upper occlusal view, (F) lower occlusal view, (G) left lateral view, (H) frontal view, and (I) right lateral view.

The initial panoramic radiograph obtained in January 2023 indicated an increased risk of bilateral ectopic eruption of the maxillary permanent canines. Eruption‐risk assessment was based on the variables described by Ericson and Kurol, including canine inclination, mesiodistal sector position, and vertical position. Tooth 1.3 presented an *α*‐angle of 44.5° and was classified as Sector 2, whereas Tooth 2.3 showed an *α*‐angle of 47.1° and was classified as Sector 3, indicating a less favorable eruptive position (Figure 4). The vertical position of both canine crowns relative to the occlusal plane was also considered qualitatively on the pretreatment panoramic radiograph as part of the overall risk assessment, although it was not used as a quantitative longitudinal outcome because of the limitations associated with linear measurements on panoramic radiographs.

**Figure 4:**
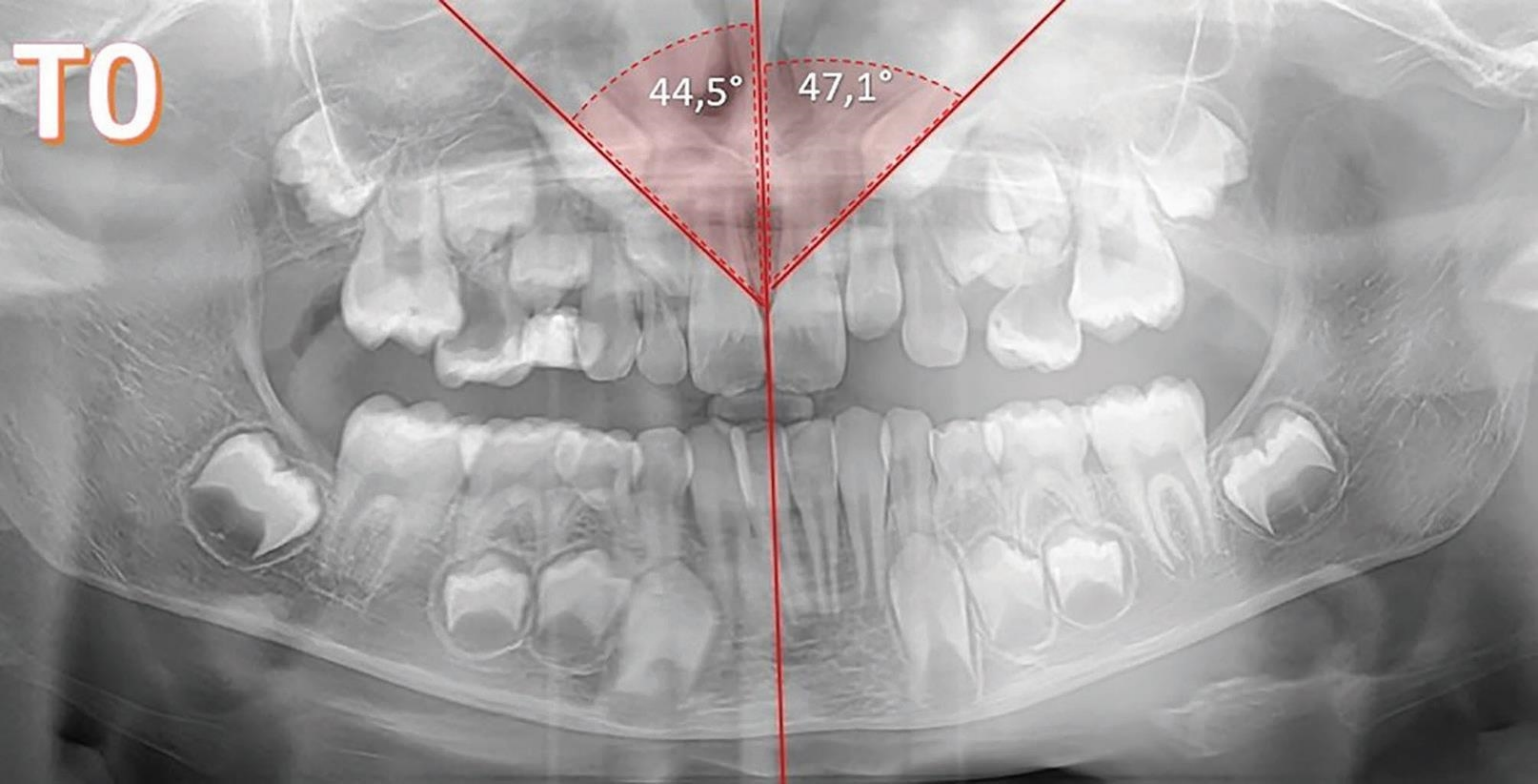
Initial panoramic radiograph at T0 showing bilateral ectopic eruption of the maxillary permanent canines.

The treatment objective was therefore not limited to space creation but also included correction of the transverse discrepancy, posterior arch coordination, preservation and redistribution of arch perimeter, molar derotation, and improvement of the eruptive environment of the maxillary permanent canines.

The treatment objectives were defined as follows:

1. Correction of the maxillary transverse deficiency.
2. Improvement and preservation of maxillary arch perimeter.
3. Derotation and distalization of the maxillary first permanent molars to improve posterior arch coordination and sagittal space availability.
4. Creation and maintenance of an adequate eruptive environment for the ectopically positioned maxillary permanent canines.
5. Improvement of the bilateral Class II molar relationship while avoiding direct orthodontic traction of the canines.

The subsequent treatment objective was therefore not limited to space creation but also included optimisation of the eruptive environment through transverse correction, posterior arch coordination, maintenance of arch perimeter, molar derotation, and sequential distalization.

### 2.2. Treatment Alternatives

The conventional alternatives discussed with the family included extraction of the primary canines alone, rapid maxillary expansion followed by headgear, and skeletal anchorage‐supported distalization using palatal miniscrews. The family refused both cervical headgear and skeletal anchorage‐supported mechanics. Because the patient presented a true transverse skeletal discrepancy, aligners alone were not considered sufficient for transverse correction. Consequently, conventional rapid maxillary expansion was selected as the first treatment phase.

### 2.3. Treatment Progress

The first phase of treatment consisted of rapid maxillary expansion to address the transverse skeletal deficiency. A hyrax‐type rapid maxillary expander cemented on first permanent molars was delivered and activated twice daily for 10 days until the desired transverse correction was clinically achieved.

Following completion of active expansion, the hyrax appliance was retained passively in situ to permit stabilization of the transverse correction and was removed immediately before initiation of the clear aligner–based sequential distalization phase. After completion of rapid maxillary expansion, an intraoral scan for aligner fabrication was obtained (October 2023). The amount of distalization achieved during treatment was assessed by superimposition of the digital intraoral scans. Figure 5 shows intraoral photographs obtained after completion of rapid maxillary expansion, documenting the postexpansion transverse arch relationship before the subsequent treatment phase.

**Figure 5:**
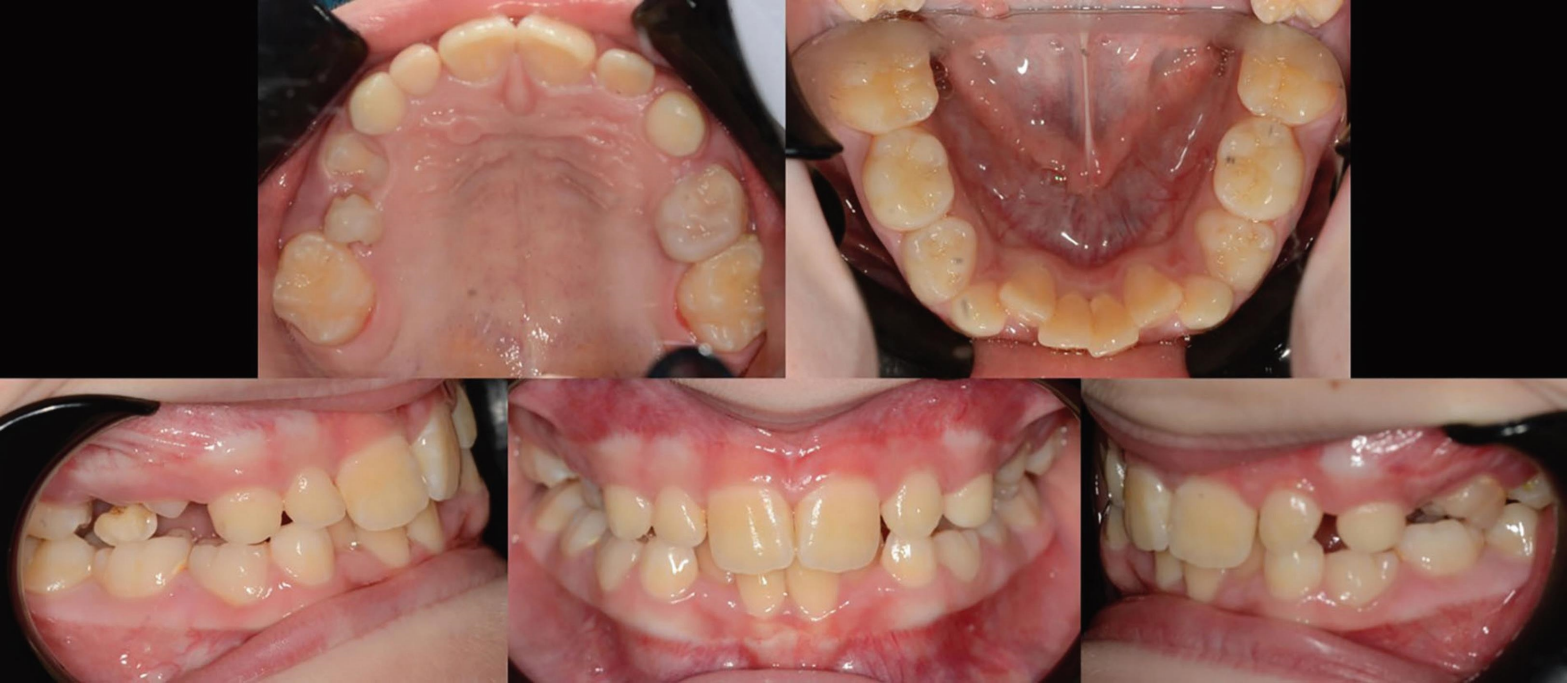
Intraoral photographs obtained after completion of rapid maxillary expansion, showing the transverse correction achieved before initiation of clear aligner therapy.

Treatment then proceeded with Invisalign FIRST therapy using a sequential distalization protocol. The digital setup included posterior sequential distalization associated with molar derotation, progressive arch coordination, maintenance and redistribution of arch perimeter, and correction of the Class II molar relationship. For the permanent first molars, transverse expansion and mesial‐out derotation were planned. Additional buccal root torque was prescribed for the maxillary posterior teeth, particularly the first permanent molars, at values 20% greater than those generated by the standard G8 setup. The biomechanical purpose was to improve buccolingual root control and counteract uncontrolled buccal crown tipping during posterior expansion and sequential distalization. The 20% increase represented an individualized clinical overcorrection selected for this patient and should not be interpreted as a universally validated or protocol‐specific threshold. A 50% sequential distalization protocol was selected in order to improve anchorage control and reduce the amount of simultaneous movement expressed by each aligner. In addition, digital eruption compensators were progressively incorporated into the aligner setup during the transition phases of the mixed dentition. Particular attention was dedicated to maintaining eruption space during physiologic premolar eruption and, subsequently, after extraction of the primary canines. These compensatory digital features were intended to preserve arch coordination and reduce undesirable space loss while maintaining adequate space for the erupting permanent canines.

Aligners were initially changed every 7 days, consistent with commonly used wear schedules [14]. During treatment, progressive aligner wear, deformation, and occasional fracture were observed despite satisfactory seating and tracking. For this reason, the replacement interval was subsequently shortened to 5 days as an individual clinical adjustment intended to maintain appliance integrity and treatment continuity. This modification was not introduced as a biomechanical component of canine eruptive guidance, and no inference regarding its effect on tooth‐movement efficiency or canine eruption can be made from this case.

Unlike traditional interceptive canine protocols, the primary canines were intentionally maintained during the distalization phase. Extraction of the primary canines was intentionally postponed until approximately 14 months after initiation of clear aligner therapy and 2 months before completion of the active distalization phase (T2), in order to preserve anchorage during active distalization. No direct traction mechanics were applied to the impacted canines during treatment. The treatment objective was therefore not direct orthodontic traction but management of the eruptive environment through a combination of transverse correction, distalization, molar derotation, arch coordination, eruption‐space preservation, and maintenance of arch perimeter.

### 2.4. Treatment Protocol

Sequential distalization was performed using an asymmetric 50% staging protocol. A total distalization of 2.6 mm was programmed for Tooth 1.6, comprising 1.8 mm during the initial aligner sequence and an additional 0.8 mm during refinement, whereas 4.0 mm was programmed for Tooth 2.6, including 3.0 mm during the initial sequence and 1.0 mm during refinement. Based on digital scan superimposition, the achieved distal movements were approximately 2.0 mm for Tooth 1.6 and 3.0 mm for Tooth 2.6. The digital setup also incorporated distal derotation of 28.7° for Tooth 1.6 and 22.7° for Tooth 2.6, together with 3.0° and 7.1° of buccal root torque, respectively. Because a greater amount of distal movement was required on the left side, distalization was initiated with Tooth 2.6; once approximately 50% of its programmed distal movement had been achieved, distalization of Tooth 1.6 was initiated while the anterior segment from canine to canine was maintained passive in the sagittal dimension to reinforce anchorage. After completion of first‐molar distalization, the premolars were sequentially distalized by approximately 1.0 mm each. Distal translation and derotation were staged at approximately 0.1 mm and 1.2° per aligner, respectively. Vertical rectangular 4‐mm attachments were placed on Teeth 1.6 and 2.6, 3‐mm horizontal rectangular attachments on Teeth 5.3, 6.3, 1.1, and 2.1, and optimized attachments on Teeth 1.2 and 2.2. In the mandibular arch, optimized attachments were used on all teeth except 3.6 and 4.6, which received horizontal rectangular attachments. Class II elastics (1/4‐inch, 4.5 oz) were introduced only during the refinement phase and worn for approximately 4 months, extending from distal buttons on Teeth 3.6 and 4.6 to hooks on Teeth 5.3 and 6.3. The initial treatment plan comprised 55 aligners; however, refinement records were obtained after Aligner 41, followed by 24 additional aligners, resulting in a total of 65 aligners actually used. The active distalization phase lasted approximately 12 months.

### 2.5. Treatment Outcomes

Progressive radiographic improvement in canine inclination and sector position was observed throughout treatment. Table 2 shows the difference in canine positions during the treatment.

At T1, following rapid maxillary expansion, an early improvement in canine position was already evident. The *α*‐angle decreased from 44.5° to 31.2° for Tooth 1.3 and from 47.1° to 31.9° for Tooth 2.3. Sector classification remained unchanged at Sector 2 for Tooth 1.3, whereas Tooth 2.3 improved from Sector 3 to 2. Clinically, post‐RME intraoral photographs also documented improvement in the transverse maxillary arch relationship before initiation of sequential distalization.

The panoramic radiographic reassessment performed at T2, approximately 16 months after T1 and following completion of the active distalization phase, showed a marked reduction in *α*‐angle values compared with baseline measurements. Notably, Tooth 2.3 initially classified as Sector 3, which was associated with the least favorable prognosis according to the Ericson and Kurol criteria, progressively exhibited a more favorable eruptive trajectory over time.

A substantial radiographic improvement was already evident before extraction of the primary canines was performed. Approximately 4 months after completion of the active distalization phase (T2) and 6 months after extraction of the primary canines, the cusp tips of both maxillary permanent canines became clinically visible in the oral cavity, confirming eruption.

The final panoramic radiograph, obtained 12 months after T2, confirmed eruption and progressive alignment of both permanent canines within the dental arch. Figures 4, 6, 7, and 8 illustrate the radiographic progression of the case. Compared with the pretreatment panoramic radiograph (Figure 4), the final radiograph (Figure 8) showed a marked improvement in the position of both maxillary permanent canines, with reduction of their mesial inclination, progression from Sectors 2 and 3 to Sector 1 for Teeth 1.3 and 2.3, respectively, and eruption of both canines into the dental arch. Posttreatment extraoral and intraoral clinical photographs are shown in Figure 9.

**Figure 6:**
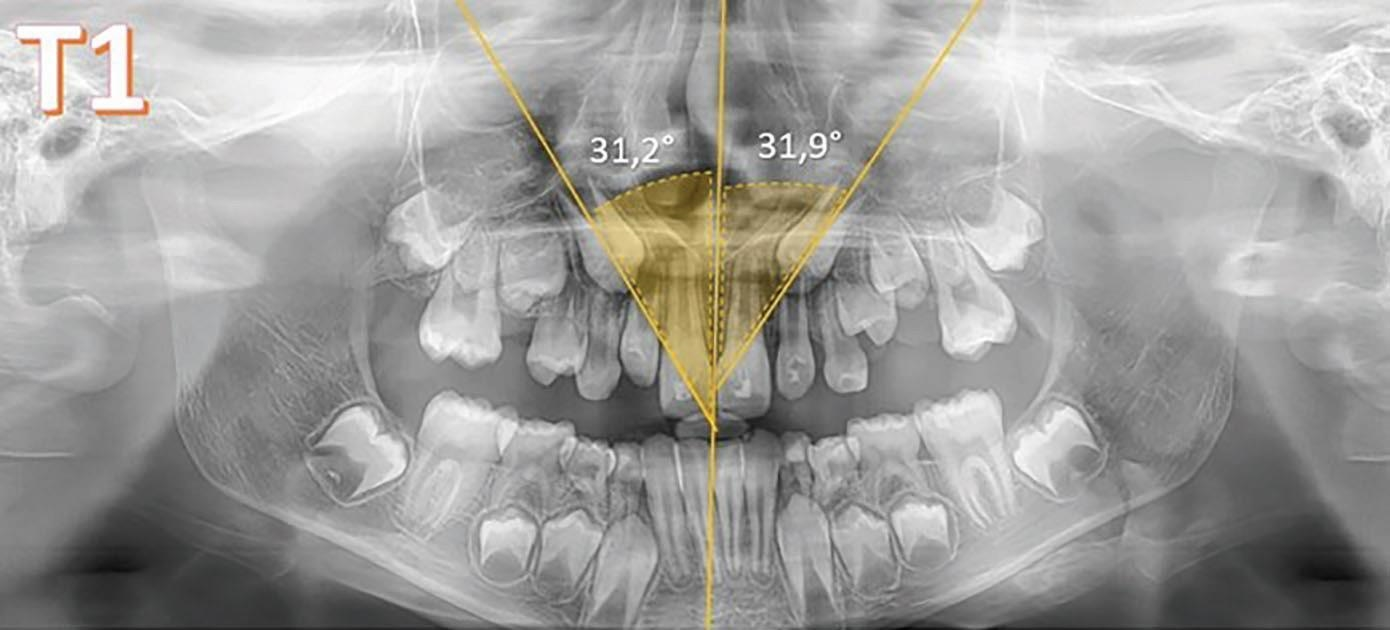
Panoramic radiograph obtained after rapid maxillary expansion (T1), showing partial improvement of canine inclination and eruption trajectory.

**Figure 7:**
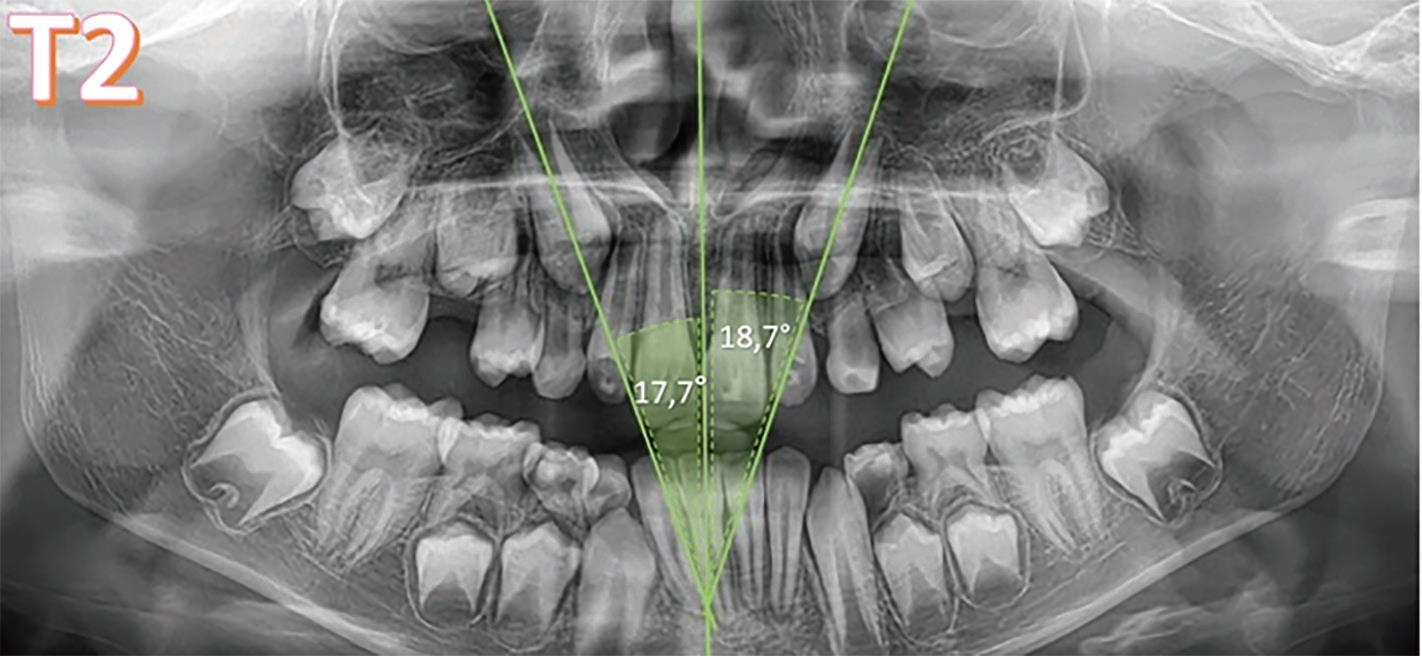
Panoramic radiograph after sequential distalization with clear aligners (T2), showing substantial reduction of *α*‐angle values and improvement of canine eruption trajectory.

**Figure 8:**
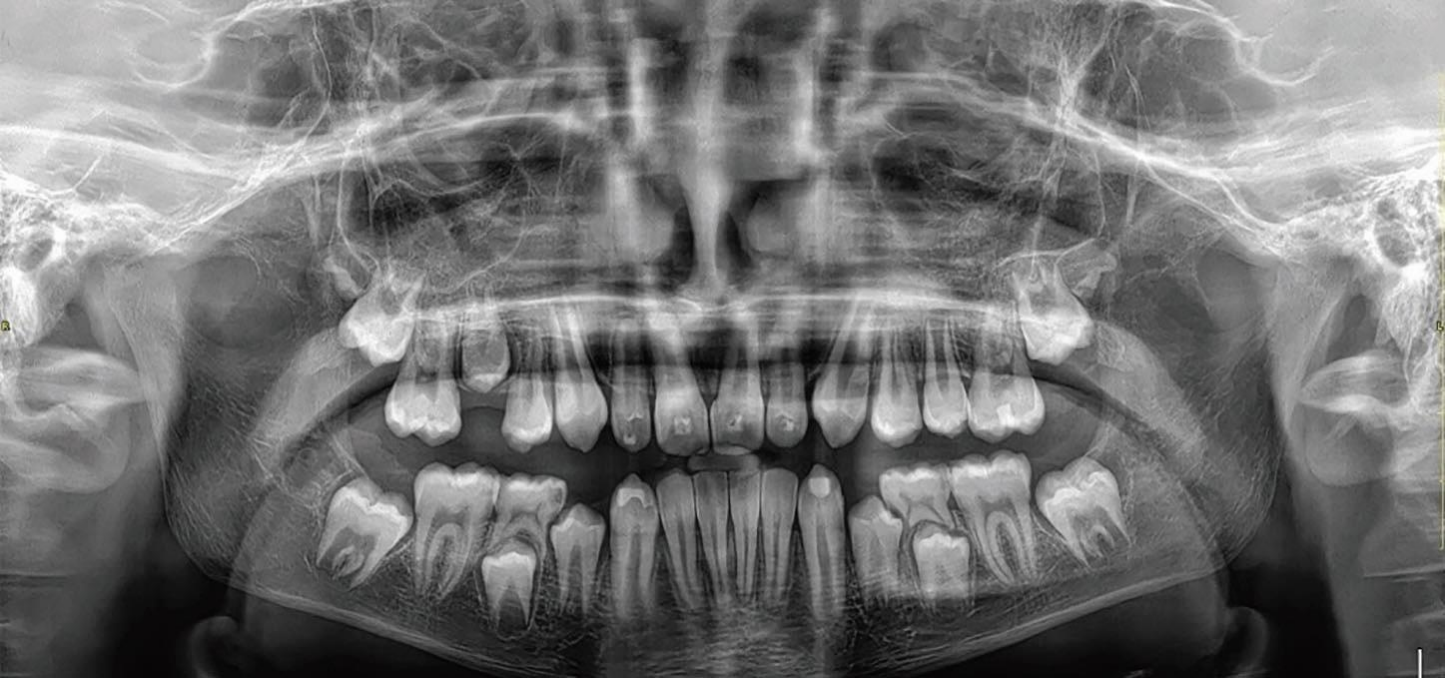
Posttreatment radiographic follow‐up showing spontaneous eruption and progressive alignment of both maxillary permanent canines.

**Figure 9:**
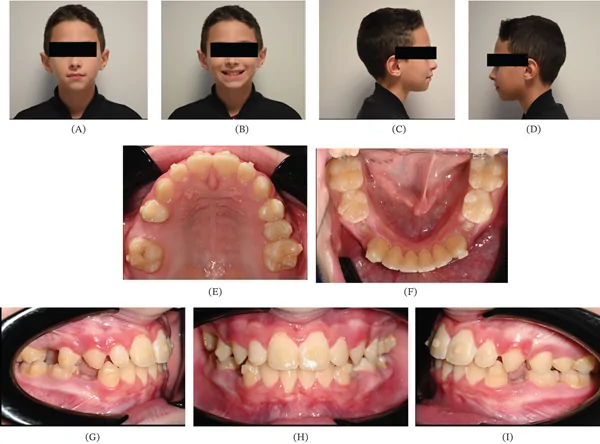
Posttreatment extraoral and intraoral photographs: (A) frontal view with lips at rest, (B) frontal view during smiling, (C) right profile view, and (D) left profile view. Intraoral photographs: (E) upper occlusal view, (F) lower occlusal view, (G) left lateral view, (H) frontal view, and (I) right lateral view.

## 3. Discussion

The present case report describes the clinical application of the BIG‐C therapeutic concept in a growing patient with bilateral ectopic eruption of the maxillary permanent canines. In this case, the approach combined rapid maxillary expansion, sequential distalization with clear aligners, and coordinated arch‐perimeter management. Accordingly, the proposed contribution of the BIG‐C concept lies in the structured integration and sequencing of established therapeutic components rather than in the introduction of a new appliance or isolated biomechanical procedure. The rationale originated from previous studies suggesting that canine eruptive guidance depends not only on extraction of the primary canine but also on maintenance or increase of upper arch perimeter [15].

In the present case report, the initial radiographic prognosis was unfavorable, particularly for Tooth 2.3. Progressive reductions in *α*‐angle and improvements in sector position were observed throughout treatment, with the largest numerical changes occurring between T1 and T2. This interval coincided with sequential distalization, the residual effects of rapid maxillary expansion and increased transverse arch dimension, molar derotation, arch‐perimeter preservation, extraction of the primary canines, physiological eruption and dental development, and ongoing craniofacial growth.

Therefore, the observed radiographic improvement cannot be attributed independently to sequential distalization. Rather, the clinical evolution should be interpreted as occurring within a multimodal interceptive protocol in which several biomechanical, developmental, and treatment‐related factors may have acted concurrently. In this context, Lugli et al. [16] described the use of clear aligners within an interceptive approach for displaced maxillary canines, combining dentoalveolar expansion and space management to support favorable eruptive conditions.

Sequential distalization represented one component of the overall treatment strategy and was intended to contribute to sagittal space management, posterior anchorage control, and staged movement of the posterior segments. Digitally programmed eruption compensators were also used to preserve eruption space and arch coordination during the transitional mixed‐dentition phases. The additional buccal root torque incorporated into the posterior setup was intended to improve root control during transverse and sagittal movement and to limit unwanted buccal crown tipping, a recognized limitation of posterior expansion with clear aligners. However, the magnitude of the overcorrection used in this patient was individually prescribed and cannot be generalized from a single case.

However, the relative contribution of these procedures to the observed canine eruption cannot be isolated from that of rapid maxillary expansion, physiological growth and dental development, primary canine management, and the other components of treatment.

The biological interpretation of the observed changes should also remain cautious. The eruption trajectory of ectopically positioned canines may be influenced by multiple concurrent factors, including available arch space, transverse maxillary development, physiological craniofacial growth, dental maturation, primary canine management, and changes in the surrounding dentoalveolar environment [17]. In the present case, the favorable clinical evolution was temporally associated with the combined interceptive protocol; however, the contribution of sequential distalization or any other individual component cannot be determined independently.

Compared with conventional headgear, clear aligners may offer several patient and clinician‐related advantages in selected cases, including improved esthetics, potentially reduced psychosocial burden, digitally planned staging of tooth movement, and simplified management of sequential or asymmetric movements. These advantages should be interpreted in light of case selection, patient compliance, and the biomechanical limitations of aligner therapy [18, 19].

## 4. Limitations of the Study

Several limitations should be considered when interpreting this case report. First, multiple treatment‐related and physiological factors acted concurrently, including rapid maxillary expansion, sequential distalization, molar derotation, arch‐perimeter management, preservation and subsequent extraction of the primary canines, craniofacial growth, and dental development. Consequently, the contribution of any individual component cannot be isolated, and the observed improvement in canine position and spontaneous eruption cannot be causally attributed to sequential distalization alone. In addition, spontaneous changes related to growth, dental maturation, and physiological eruption cannot be excluded. For the same reason, vertical canine displacement relative to the occlusal plane was not quantified longitudinally, and the radiographic outcome assessment was therefore primarily based on *α*‐angle and sector classification. Second, assessment of canine position was based on serial panoramic radiographs, which provide only two‐dimensional information and are subject to projection‐related distortion and magnification. Three‐dimensional assessment with cone‐beam computed tomography was not performed; therefore, buccopalatal position, root morphology, proximity to adjacent roots, and three‐dimensional changes in eruption trajectory could not be quantified. However, CBCT was not considered justified solely for research purposes in this young patient because of the additional radiation exposure.

The findings should also be interpreted within the context of the specific clinical characteristics of this patient. The biomechanical rationale underlying the BIG‐C concept may be relevant to selected patients presenting with a Class II molar relationship, potential for posterior distalization, and the possibility of increasing sagittal arch perimeter; however, its clinical applicability and reproducibility cannot be established from a single case. Further studies involving larger samples, appropriate comparison groups, longitudinal follow‐up, and three‐dimensional imaging when clinically justified are needed to determine the relative contribution of the individual treatment components and to further evaluate this therapeutic approach.

## 5. Conclusion

Within the limitations of a single case report, application of the combined interceptive approach, including rapid maxillary expansion, sequential distalization with clear aligners, and arch‐perimeter management, was associated with progressive reduction in *α*‐angle values, improvement in sector classification, and eruption of both maxillary permanent canines without surgical exposure or direct orthodontic traction. Because several treatment‐related and physiological factors acted concurrently, including rapid maxillary expansion, growth and dental maturation, primary canine extraction, and sequential distalization, the present case cannot establish the independent contribution of any single treatment component. Controlled studies with larger samples and three‐dimensional assessment are needed to further evaluate the reproducibility and clinical relevance of this approach.

## Author Contributions

Conceptualization, G.I., D.L., and L.B.; methodology, M.L. and D.C.; investigation, E.G.; data curation, F.E. and M.T.; writing—original draft preparation, M.L.; writing—review and editing, M.L. and L.L.R.; supervision, L.B. and L.L.R.

## Funding

No funding was received for this manuscript.

## Conflicts of Interest

The authors Daniela Lupini, Gaetano Iluzzi, and Lorenzo Bartolommei have educational and/or mentoring relationships with Align Technology. These relationships had no role in the clinical management of the patient, study design, data collection, data interpretation, manuscript preparation, or the decision to submit the article for publication.

## Data Availability

The data that support the findings of this study are available from the corresponding author upon reasonable request.
